# Dietary supplementation of Galactooligosaccharides improves growth performance and intestinal barrier function of offspring

**DOI:** 10.1186/s40813-025-00437-6

**Published:** 2025-07-04

**Authors:** Jichang Zheng, Haibo Shen, Bi’e Tan, Yulong Yin, Jing Wang

**Affiliations:** 1 Hunan Provincial Key Laboratory for the Products Quality Regulation of Livestock and Poultry, College of Animal Science and Technology, Hunan Agricultural University, Changsha, 410128 Hunan China; 2 Yuelushan Laboratory, Changsha, 410128 China

**Keywords:** Ningxiang piglets, Maternal supplementation, Galactooligosaccharides, Intestinal barrier, Microbiota

## Abstract

This study aims to investigate the effects of maternal supplementation with galactooligosaccharides (GOS) on intestinal digestion, absorption, and barrier function of offspring. Twenty four late-pregnant Ningxiang sows were randomly assigned into 3 treatments, including basic diet, basic diet with 0.5% GOS, basic diet with 100 mg/kg aureomycin, 20mg/kg ciprofloxacin and 10mg/kg vancomycin. The results showed that maternal dietary supplementation of GOS increased the body weight and average daily gain, reduced the diarrhea score of piglets, and increased the activities of digestive enzymes including maltase and α-amylase. Maternal GOS improved the intestinal barrier integrity and function of piglets, which exhibiting with increased ileal ratio of villus to crypt, and higher expressions of secretory immunoglobulin A, tight junction protein and mucin-2 in ileum. Maternal GOS treatment increased the contents of anti-inflammatory factors IL-4, IL-10 and TGF-β, but decreased the contents of pro-inflammatory factors IL-1β, IL-6 in the ileum and colon of piglets. In addition, piglets in maternal GOS treatment had increased abundance of probiotics in the colonic contents, as well as higher short-chain fatty acids concentrations in the colonic and cecal contents. Overall, this study provides data support for the nutritional regulation of maternal supplementation with 0.5% GOS to promote intestinal health of offspring.

**Graphical abstract:**

Maternal dietary supplementation with GOS can enhance the intestinal health of piglets 
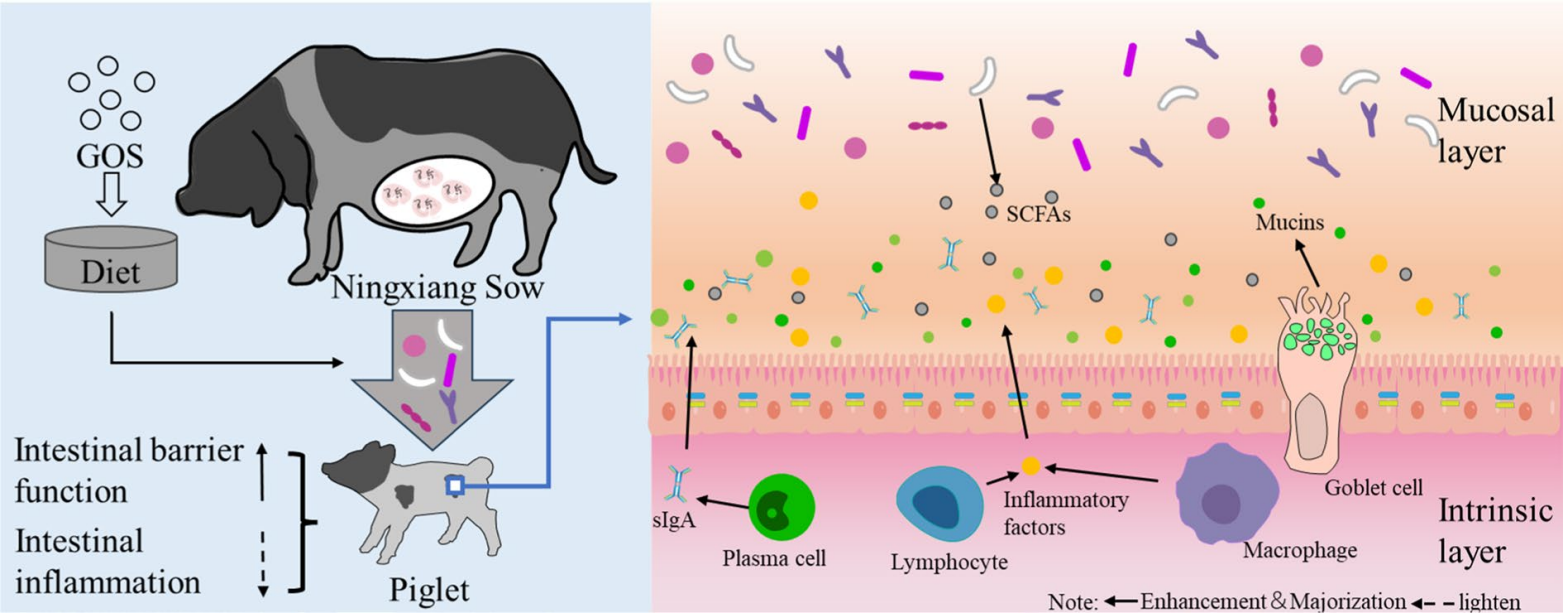

## Highlights

1. This study provides data support for the dietary fiber nutrition regulation for the integration of mother and offspring.
2. Maternal GOS supplementation can improve the growth performance of offspring and alleviate diarrhea
3. Maternal GOS supplementation can increase the richness of *Ruminococcaceae* and increase the expression of short-chain fatty acids such as propionic acid and butyric acid in the intestine.

## Introduction

A balanced and healthy diet has increasingly become a mainstream pursuit in contemporary society, with dietary fiber emerging as a focal point of interest. Dietary fiber is not digestible or absorbable in the gastrointestinal tract for energy production and is classified into two categories based on its solubility: soluble dietary fiber, which serves as a substrate for microbial metabolism in the gut, and insoluble dietary fiber, which aids in gastrointestinal motility and promotes digestion and absorption [12, 15]. Dietary fiber has been proven to play a pivotal role in maintaining host homeostasis and health, concurrently optimizing gut microbiota composition and effectively mitigating excessive weight gain and constipation issues during pregnancy [11, 16]. Moreover, studies have demonstrated that the supplementation of dietary fiber during pregnancy can alleviate metabolic disorders, such as gestational hypertension and gestational diabetes, which are associated with adverse birth outcomes [30, 41]. However, the influence of maternal dietary fiber supplementation on the developmental outcomes of infants and young children remains an area of ongoing research requiring further exploration.

During infancy, the gastrointestinal tract is underdeveloped, and due to rapid growth and high nutritional demands, it often operates under excessive strain, leading to symptoms such as indigestion, abdominal pain, and diarrhea, indicative of gastrointestinal dysfunction [8, 54]. The gestational and lactation periods represent critical windows for intestinal development in infants; enhancing maternal health during pregnancy and lactation, strengthening maternal nutrient transfer, and improving the intestinal barrier function of neonates are optimal strategies for maintaining gut health [4, 18]. Due to ethical concerns regarding animal research, studies frequently employ the "sow-piglet" model [58]. For instance, the supplementations of fermented bamboo fiber to sows during late gestation and lactation periods enhance the relative abundance of gut probiotic in offspring, and simultaneously reducing the concentration of the pro-inflammatory factor Interleukin-6 (IL-6) in the serum of both sows and piglets [40]. Moreover, supplementations of wheat bran and beet pulp during late gestation and lactation increase the abundance of acidophilic bacteria, and elevate the concentration of ileum secretory immunoglobulin A (sIgA), while reducing the concentration of the pro-inflammatory factor Tumor Necrosis Factor-α (TNF-α) in the ileum of piglets [36].

As one of the four renowned pig breeds in China, Ningxiang pigs have historically consumed a high-fiber diet, resulting in a distinctive gut microflora [21]. Soluble dietary fiber supplement Galactooligosaccharides (GOS), has previously been shown to be an effective prebiotic that positively regulates the gut microbiota, increases beneficial bacteria, and maintains intestinal health when directly supplemented to piglets [10, 49]. However, the transgenerational effects of maternal GOS intervention on offspring intestinal development and its underlying mechanisms remain incompletely understood. Given that antibiotics are known to disrupt gut microbiota and impair intestinal health, they can serve as a negative control [19, 35]. In this study, we innovatively propose that maternal GOS supplementation regulates the microbial colonization dynamics of offspring. Using Ningxiang sows and piglets as the research model, we established a comparative experimental design with a control treatment, a GOS treatment as a positive modulator, and an antibiotic treatment as a negative modulator. This study systematically explores the transgenerational effects of maternal GOS supplementation on the growth performance, intestinal barrier function, and microbial structure of piglets.

## Materials and methods

### Animals and experimental design

Twenty four late-pregnant Ningxiang sows, with parity ranging from 2 to 4, were randomly assigned to three experimental treatments (8 sows per treatment): Control (Con), Galactooligosaccharides (GOS), and Antibiotic (AB) treatments. The trial period extended from gestational day 109 to lactation day 24, encompassing 31 days. Piglets in the Con treatment received a standard basal diet. The GOS treatment received the basal diet supplemented with 0.5% GOS by feed weight (approximately 70% purity), serving as a prebiotic intervention to positively modulate the gut microbiota. The AB treatment received the basal diet supplemented with 100 mg/kg chlortetracycline, 20 mg/kg ciprofloxacin, and 10 mg/kg vancomycin, serving as a microbiota interference treatment with negative modulation. Post-experiment, eight healthy piglets were randomly selected from each treatment (one male piglet every litter) for slaughter and sampling.

Throughout the experiment, sows were fed twice daily at 7:00 and 15:00 according to the pig farm's requirements. All nutrients in the basal diet met NRC (2012) guidelines (Table 1). From Day 109 of gestation until the day prior to parturition, each sow was fed an average of 3 kg/d of feed. On the day of parturition, sows were provided with 1.0 kg of daily ration, which was then incrementally increased by 0.8 kg on the second and third days postpartum, followed by a daily increase of 1.0 kg from the fourth day onward, until reaching the maximum feed intake. Sows had free access to water. The daily intake of GOS and antibiotics by sows is calculated based on 3 kg of feed to ensure an intake of approximately 10 g GOS/day per sow.

**Table 1:** Nutrient level of basic diet (Air-dry foundation)

Ingredients or Nutrient level	Content
Corn (%)	61.00
Sanctuary (%)	10.00
Wheat bran (%)	8.00
Soybean lees (%)	17.00
Limestone (%)	0.50
Calcium bicarbonate	1.50
Premix^①^ (%)	2.00
Total (%)	100
Digestible Energy, DE (MJ/kg)	16.63
Crude Protein, CP	15.10
Crude Fat, EE	10.10
Crude Fiber, CF	9.17
Nitrogen Free Extract, NFE	38.23
Lysine (%)	0.82
Calcium (%)	0.68
Available phosphorus (%)	0.32
Methionine + Cystine (%)	0.51

① Premier provides food feed per kg of food: Vitamin A 2000 IU, Vitamin D 200 IU, Vitamin E 45 IU, Vitamin K 0.5 mg, Vitamin B_1_ 1 mg, Vitamin B_6_ 3.85 mg, Vitamin B_12_ 15 mg, pantotide, pantothenic acid 12 mg, 10.25 mg of niacin, 1.35 mg of folic acid, 0.21 mg of biology, Mn(MnSO_4_) 20 mg, Fe(FeSO_4_) 100 mg, Cu(CuSO_4_) 20 mg, I(CaI_2_) 0.14 mg, Se(Na_2_SeO_3_) 0.15 mg;

② The nutrition level is the theoretical calculation value

Sows were transferred to the delivery room one week before the expected delivery. The temperature of the delivery room was strictly maintained at 20 °C, with lighting controlled from 06:00 to 16:00. Lactating piglets were allowed to suckle freely and were managed according to routine farm practices. The study was conducted at the Yongxing Pig Farm of Hunan Chuweixiang Agriculture & Animal Husbandry Co., LTD. (Changsha 410,000, China).

Various parameters, including litter size, neonatal piglet health status, and piglet weights at birth and on days 3, 7, 14, and 21 of lactation, were recorded. Additionally, the reproductive performance of sows and the average daily gain (ADG) of piglets were calculated and analyzed.

$${\text{ADG }}\left( {{\text{g}}/{\text{day}}} \right) = \left( {{\text{d21}}\;{\text{weight}}{-}{\text{d3}}\;{\text{weight}}} \right)/{18}$$

### Diarrhea index of suckling piglets

Fecal morphology was recorded for piglets on the 3rd, 7th, 14th, and 21st days of lactation. Rectal fecal samples were collected using anal swabs, and the fecal consistency was scored according to the established fecal score standard (Table 2) to calculate the diarrhea score for each piglet.

**Table 2:** The standard of diarrhea score

Diarrhea degree	Excrement shape	Diarrhea score
Normal diarrhea	Normal feces	0.00
Mild diarrhea	Soft feces (partially formed)	1.00
Moderate diarrhea	Semi liquid state (Pasty feces)	2.00
Severe diarrhea	Water sample feces	3.00

### Sample collection

After weighing, piglets undergo humane euthanasia (anesthesia followed by jugular vein puncture), and blood samples were collected from the anterior vena cava. Three tubes containing serum and plasma were obtained from each blood collection vessel. Following a 30-min incubation at room temperature, the samples were centrifuged at 3000 rpm for 10 min. The supernatant was subsequently aliquoted into 1.5 ml centrifuge tubes and stored at – 80 °C to prevent hemolysis. Additionally, fixed, molecular, and mucosal samples were collected from the ileum and colon, along with chyme from the colon and cecum, for subsequent experimental analyses.

### Indicator detection

#### Organ index

During the dissection of piglets, a scalpel was used to sequentially excise the heart, liver, spleen, lungs, kidneys, and thymus. The residual blood on the surface of the organs was carefully wiped off, and each organ was weighed three times, with the average weight recorded for statistical analysis.

The organ index was calculated using the following formula: Organ index (%) = organ weight / piglet body weight *100%

#### Gastrointestinal pH

During the dissection of piglets, a pH meter (Sartorius, Germany) was used to measure the pH levels in the stomach, cecum, and colon.

#### Ileal digestive enzyme activity

The activities of sucrase, maltase, amylase, lipase, and trypsin in the ileum of piglets were assessed using a reagent kit (Nanjing Jiancheng Biotechnology Research Institute).

#### Morphometric measurement of ileum tissue

Intestinal samples were collected, and histological morphology was examined using hematoxylin and eosin (H&E) staining. The samples underwent dehydration, embedding, sectioning, and subsequent staining with H&E. Villus height and crypt depth were measured using a computer-aided microscope (ZEISS Axioscope 5, Germany), and the villus-to-crypt ratio (villus height/crypt depth) was calculated.

#### Serum biochemical indicators and levels of inflammatory factors

Diamine oxidase (DAO) and D-lactic acid (D-LA) levels in the serum of piglets were measured using a reagent kit (G0154W, G0827W, Suzhou Grace Biotechnology Co.,Ltd). The concentrations of inflammatory factors in the serum, including Interleukin-2 (IL-2), Interleukin-4 (IL-4), IL-6, Interleukin-8 (IL-8), Interleukin-12 (IL-12), and TNF-α, were determined using Luminex detection technology.

#### Ileal secretory immunoglobulin A content

Secretory immunoglobulin A (sIgA) levels in the ileum were determined using enzyme-linked immunosorbent assay kits (CSB-E112063p, CUSABIO, https://www.cusabio.com/) according to the manufacturer's instructions.

#### Immunofluorescence

Tissue sections were deparaffinized and rehydrated through a graded series of ethanol concentrations (100%, 100%, 95%, 85%, 75%, and water), followed by washing with PBS. The sections were then blocked with 5% BSA blocking solution at room temperature for 30 min. Primary antibodies targeting tight junction proteins (ZO-1, occludin) and mucin 2 (MUC2) (Aifang Biotechnology, Changsha, China), Immunoglobulin A (sIgA) (Proteintech, Wuhan Sanying, China), cell proliferation markers (Ki67), and inflammasomes (NLRP3) were applied and incubated overnight at 4°C. Subsequently, sections were incubated with goat anti-rabbit IgG secondary antibody conjugated with horseradish peroxidase for 1 h at room temperature. The sections were stained with 6-diamino-2-phenylindole (DAPI) for 10 min. Finally, the sections were cleared and mounted with a sealing solution containing an anti-fluorescence quencher. Images were observed and captured using a fluorescence microscope (Zeiss, Germany).

#### Real-time PCR

According to primer design principles, primers were designed using Primer Premier 6.0 software and synthesized by Beijing Tsingke Biotech Co., Ltd. and Sangon Biotech (Shanghai) Co., Ltd. The primer sequences are listed in Table 3. Total RNA was extracted from liver tissues using TRIZOL reagent (Invitrogen, Carlsbad, CA, United States). The integrity, quality, and purity of the RNA samples were assessed by 1% agarose gel electrophoresis and an automated nucleic acid/protein analyzer. RNA was reverse-transcribed into cDNA using the Evo M-MLV Reverse Transcription Premix Kit (AG11728, Accurate Biotechnology (Hunan) Co., Ltd., Changsha, China). Real-time fluorescence quantitative PCR was performed using the SYBR Green Pro Taq HS Premixed qPCR Kit (AG11701, Accurate Biotechnology (Hunan) Co., Ltd., Changsha, China) on a LightCycler®480 Real-Time PCR System (Roche, Switzerland). The reactions were carried out in a 10 μL system, and each reaction was conducted in triplicate. The reaction conditions followed the protocol described by Qi Ming et al. β-actin was used as the internal reference gene, and the 2^-ΔΔCt method was employed to calculate the relative mRNA expression levels of the target genes.

**Table 3:** Primer sequence

Gene	Nucleotide sequence of primers (5′–3′)	GenBank accession No	Size, bp
Beta-actin-F	CTGCGGCATCCACGAAACT	XM_021086047.1	147
Beta-actin-R	AGGGCCGTGATCTCCTTCTG		
MUC2-F	CTGTGTGGGGCCTGACAA	XM_021082584.1	65
MUC2-R	AGTGCTTGCAGTCGAACTCA		
IL-1β-F	CAGCCATGGCCATAGTACCT	XM_021085847.1	216
IL-1β-R	CCACGATGACAGACACCATC		
IL-6-F	AAATGTCGAGGCCGTGCAGATTAG	NM_214399.1	86
IL-6-R	GGGTGGTGGCTTTGTCTGGATTC		
IL-4-F	CCCGAGTGTCAAGTGGCTTA	NM_214340.1	122
IL-4-R	TGATGATGCCGAAATAGCAG		
IL-10-F	GGGCTATTTGTCCTGACTGC	NM_214041.1	105
IL-10-R	GGGCTCCCTAGTTTCTCTTCC		
TGF-β-F	AGGGCTACCATGCCAATTTCT	XM_021093503.1	101
TGF-β-R	CGGGTTGTGCTGGTTGTACA		

*MUC* Mucin; *IL* Interleukin; *TGF* Transforming growth factor

#### Microbial analysis of colon contents

Using the Illumina NovaSeq sequencing platform (BMKCloud (www.biocloud.net)), a paired-end sequencing method was employed to construct a small fragment library for microbial sequencing of colon chyme. Sequence reads were concatenated, filtered, clustered or denoised, followed by species annotation and abundance analysis involving the following aspects:

1. *Alpha Diversity Analysis* The species diversity within individual samples was assessed through alpha diversity metrics, with statistical analysis of the Shannon and Simpson indices for each sample;
2. *Beta Diversity Analysis* Differences in species diversity (community composition and structure) among different samples were compared using beta diversity analysis. Principal Component Analysis (PCA) and Principal Coordinates Analysis (PCoA) plots were generated based on the distance matrix;
3. *OTU Analysis* Operational Taxonomic Unit (OTU) analysis was performed to produce Venn diagrams illustrating each treatment's sample overlap at the phylum and genus levels, along with genus-level species clustering heatmaps;
4. *Differential Bacterial Analysis* The LEfSe (Linear Discriminant Analysis Effect Size) method (LDA > 3.0) was used to measure differences in species abundance composition between treatments;
5. *Correlation Analysis* The correlation between species richness and environmental factors (e.g., short-chain fatty acid content in the colon, immunoglobulin A content, and expression of inflammatory factors) was analyzed using correlation heatmaps.

#### Short chain fatty acid content

Approximately 1 g of chyme was accurately weighed and the mass of each sample was recorded. The chyme was then homogenized with 5 mL of ultrapure water using vortex mixing and mechanical shaking for 30 min. The homogenate was incubated overnight at 4 °C, followed by centrifugation at 10,000 rpm for 10 min. The supernatant was carefully transferred, and the precipitate was resuspended in 4 mL of ultrapure water. After another 30 min of shaking, the resuspended precipitate was centrifuged, and the supernatants were pooled into a 10 mL volumetric flask. The pooled supernatant was transferred to a 10 mL centrifuge tube and centrifuged at 12,000 rpm for 15 min. The clarified supernatant was then aliquoted into a 2 mL centrifuge tube at a ratio of 9:1 (900 µL supernatant + 100 µL of 25% phosphoric acid). The mixture was thoroughly mixed and allowed to stand at room temperature for 3–4 h prior to centrifugation. The sample was subsequently filtered through a 45 µm microporous nylon filter membrane and injected into a gas chromatograph equipped with a DB-FFAP column. The concentrations of short-chain fatty acids (acetic acid, propionic acid, isobutyric acid, butyric acid, isovaleric acid, and valeric acid) in the colon and cecal chyme were quantified.

### Statistical analysis

One-way analysis of variance (ANOVA) was employed to determine significant differences between treatments. Prior to analysis, data were assessed for normality and homogeneity of variance. All data are presented as mean ± standard error of the mean (SEM). Statistical analyses were performed using SPSS version 26.0 (IBM, SPSS). A probability value (*P*-value) of less than 0.05 was considered statistically significant, while a *P*-value between 0.05 and 0.10 was interpreted as indicative of a trend among the treatments.

## Results

### Reproductive performance of sow

The reproductive performance of Ningxiang sows were shown in Table 4. GOS treatment significantly increased the number of healthy piglets as compared to the Control treatment (*P* < 0.05). GOS treatment showed a trend to decrease the number of weak piglets (*P* < 0.1). However, no significant differences were observed in the total born piglets, live born piglets, intrauterine growth restriction rate, stillbirth rate, number of mummified piglets, and litter weight at birth among three treatments.

**Table 4:** Sow reproduction

Items	Con	GOS	AB	SEM	*P***-**value
Total born piglets	9.05	9.00	8.91	1.718	0.965
Live born piglets	8.53	8.88	8.68	1.308	0.688
Number of healthy piglets	7.16^b^	8.29^a^	7.77^ab^	1.340	0.020
Number of weak piglets	1.37	0.58	0.86	1.086	0.059
Intrauterine growth restriction rate, %	14.59	9.60	9.21	16.063	0.502
Number of stillborns	0.42	0.50	0.18	0.627	0.210
Stillbirth rate, %	4.90	8.49	1.97	13.540	0.265
Number of mummified pigs	0.00	0.08	0.05	0.211	0.446
Litter weight at birth, g	959.49	954.23	948.86	139.278	0.963

Values are presented as means ± SEM, One-Way ANOVA, Different lowercase letters indicate significant differences amongst treatment treatments (*P* < 0.05). Con, Basal diet; GOS, Basic diet with 0.5% Galactooligosaccharides (Purity of approximately 70%); AB, Basic diet with 100 mg/kg chloramphenicol, 20 mg/kg ciprofloxacin, and 10 mg/kg vancomycin

### Effect of maternal GOS supplementation on growth performance of piglets

As shown in Table 5, maternal dietary supplementation with GOS significantly increased the body weight of piglets at d 14 and d 21 of age as compared to the AB treatments, and maternal GOS treatment showed the highest ADG of piglets (*P* < 0.05). Maternal GOS treatment also decreased the diarrhea score of piglets at d 14 and d 21 of age (*P* < 0.05). Additionally, the liver index and thymus index of piglets in GOS treatment were higher than those of piglets in Control treatment (*P* < 0.05).

**Table 5:** Growth performance and organ index of piglets

Items	Con	GOS	AB	SEM	*P*-value
*Growth performance*, g					
Body weight at d 3 of age	1455.65	1462.45	1422.08	265.764	0.829
Body weight at d 7 of age	2283.84	2313.37	2190.44	437.265	0.555
Body weight at d 14 of age	3868.30^ab^	4133.10^a^	3703.25^b^	604.477	0.049
Body weight at d 21 of age	5527.23^ab^	5856.48^a^	5197.37^b^	722.225	0.006
Average daily gain	219.58^b^	236.63^a^	210.40^b^	35.764	0.033
*Diarrhea score*					
Diarrhea score at d 3 of age	0.51	0.51	0.52	0.839	1.000
Diarrhea score at d 7 of age	0.47	0.34	0.55	0.803	0.569
Diarrhea score at d 14 of age	0.47^ab^	0.14^b^	0.62^a^	0.781	0.035
Diarrhea score at d 21 of age	0.38^a^	0.06^b^	0.66^a^	0.589	< 0.001
*Organ index*, g/kg					
Heart index	5.67	5.54	5.53	0.409	0.779
Liver index	21.87^b^	24.20^a^	23.61^ab^	1.861	0.043
Spleen index	2.57	2.20	2.33	0.695	0.596
Lung index	10.82	10.79	10.79	0.921	0.269
Kidney index	7.16	6.46	7.23	0.741	0.131
Thymus index	0.64^b^	0.99^a^	0.71^ab^	0.289	0.036

Values are presented as means ± SEM, One-Way ANOVA, Different lowercase letters indicate significant differences amongst treatment treatments (*P* < 0.05). Con, Basal diet; GOS, Basic diet with 0.5% Galactooligosaccharides (Purity of approximately 70%); AB, Basic diet with 100 mg/kg chloramphenicol, 20 mg/kg ciprofloxacin, and 10 mg/kg vancomycin

### Effect of maternal GOS supplementation on intestinal digestive capacity of piglets

The gastrointestinal pH levels were presented in Table 6. Maternal dietary supplementation with GOS reduced the pH levels in stomach, cecum, and colon of piglets (*P* < 0.05).

**Table 6:** The pH of the gastrointestinal tract

Items	Con	GOS	AB	SEM	*P*-value
Stomach	4.92^a^	3.60^b^	3.64^b^	0.717	< 0.001
Cecum	7.31^a^	6.55^b^	7.36^a^	0.495	< 0.001
Colon	7.20^a^	6.54^b^	7.23^a^	0.420	< 0.001

Values are presented as means ± SEM, One-Way ANOVA, Different lowercase letters indicate significant differences amongst treatment treatments (*P* < 0.05). Con, Basal diet; GOS, Basic diet with 0.5% Galactooligosaccharides (Purity of approximately 70%); AB, Basic diet with 100 mg/kg chloramphenicol, 20 mg/kg ciprofloxacin, and 10 mg/kg vancomycin

**Fig. 1:**
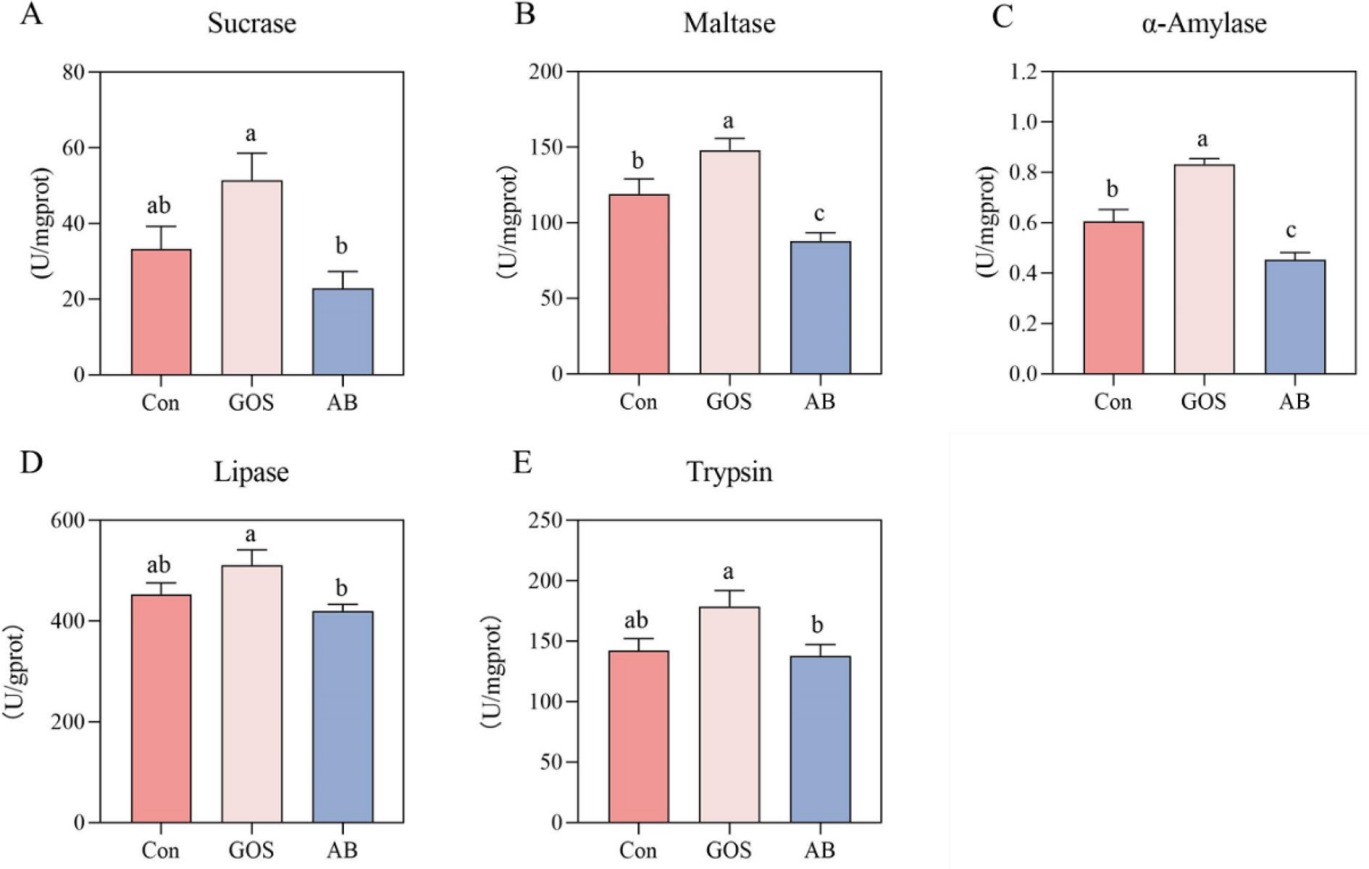
Effect of maternal GOS supplementation on digestive enzymes of piglets. **A** Sucrase, **B** Maltase, **C** α-Amylase, **D** Lipase, **E** Trypsin. Values are presented as means ± SEM, n = 8, One-Way ANOVA, Different lowercase letters indicate significant differences amongst treatment treatments (*P* < 0.05). Con, Basal diet; GOS, Basic diet with 0.5% Galactooligosaccharides (Purity of approximately 70%); AB, Basic diet with 100 ppm chloramphenicol, 20 ppm ciprofloxacin, and 10 ppm vancomycin

As illustrated in Fig. 1, the ileal maltase and α-amylase activities of piglets in GOS treatment were significantly higher than those in Con and AB treatments (*P* < 0.05). And compared to the AB treatment, maternal GOS treatment also enhanced the activities of sucrase, lipase and trypsin in ileum of piglets (*P* < 0.05).

### Effect of maternal GOS supplementation on intestinal barrier morphology and integrity of piglets

**Fig. 2:**
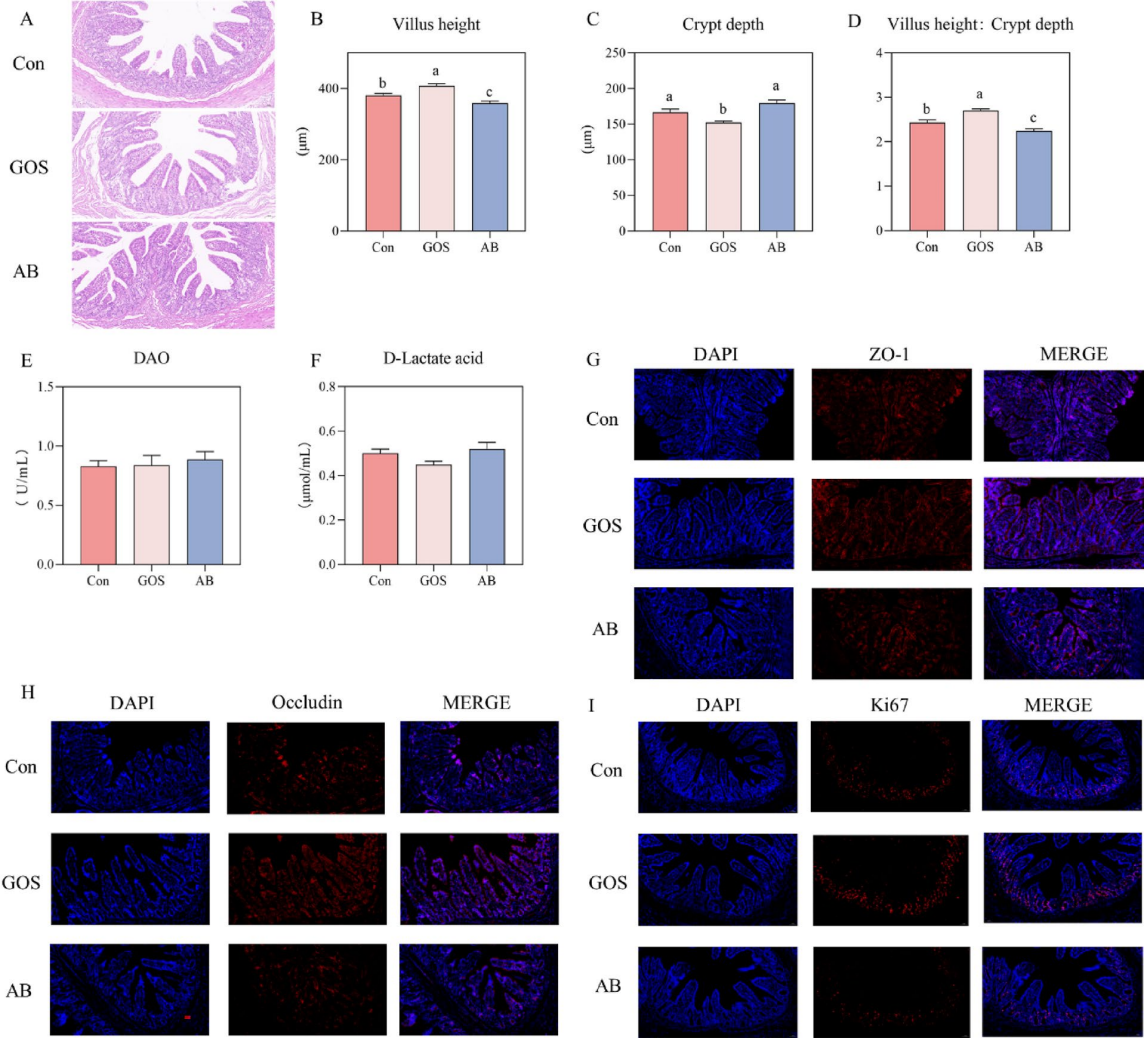
Effect of maternal GOS supplementation on the ileal morphology of piglets. **A** Hematoxylin and eosin (H&E) staining, **B** Ileal Villus height, **C** Ileal Crypt depth, **D** Ileal Villus height: Crypt depth, **E** Serum ammonium dioxide enzyme level, **F** Serum D-lactate levels, **G** Immunofluorescence of Zonula occludens 1 (ZO-1), **H** Immunofluorescence of Occludin, **I** Immunofluorescence of Ki67(scale bars: 50 μm). Values are presented as means ± SEM, n = 8, One-Way ANOVA, Different lowercase letters indicate significant differences amongst treatment treatments (*P* < 0.05). Con, Basal diet; GOS, Basic diet with 0.5% Galactooligosaccharides (Purity of approximately 70%); AB, Basic diet with 100 ppm chloramphenicol, 20 ppm ciprofloxacin, and 10 ppm vancomycin

As depicted in Fig. 2, the ileal villi height of piglets in the GOS treatment was notably higher, while the crypt depth was deeper, resulted in a larger V/C compared to those in the Con and AB treatments (*P* < 0.05). The fluorescence intensities of tight junction proteins (ZO-1, Occludin), and Ki67 were more pronounced in the GOS treatment. However, no significant differences were observed in serum DAO and D-lactic acid levels among the three treatments.

### Maternal GOS supplementation regulated intestinal inflammatory and MUC2 expression of piglets

**Fig. 3:**
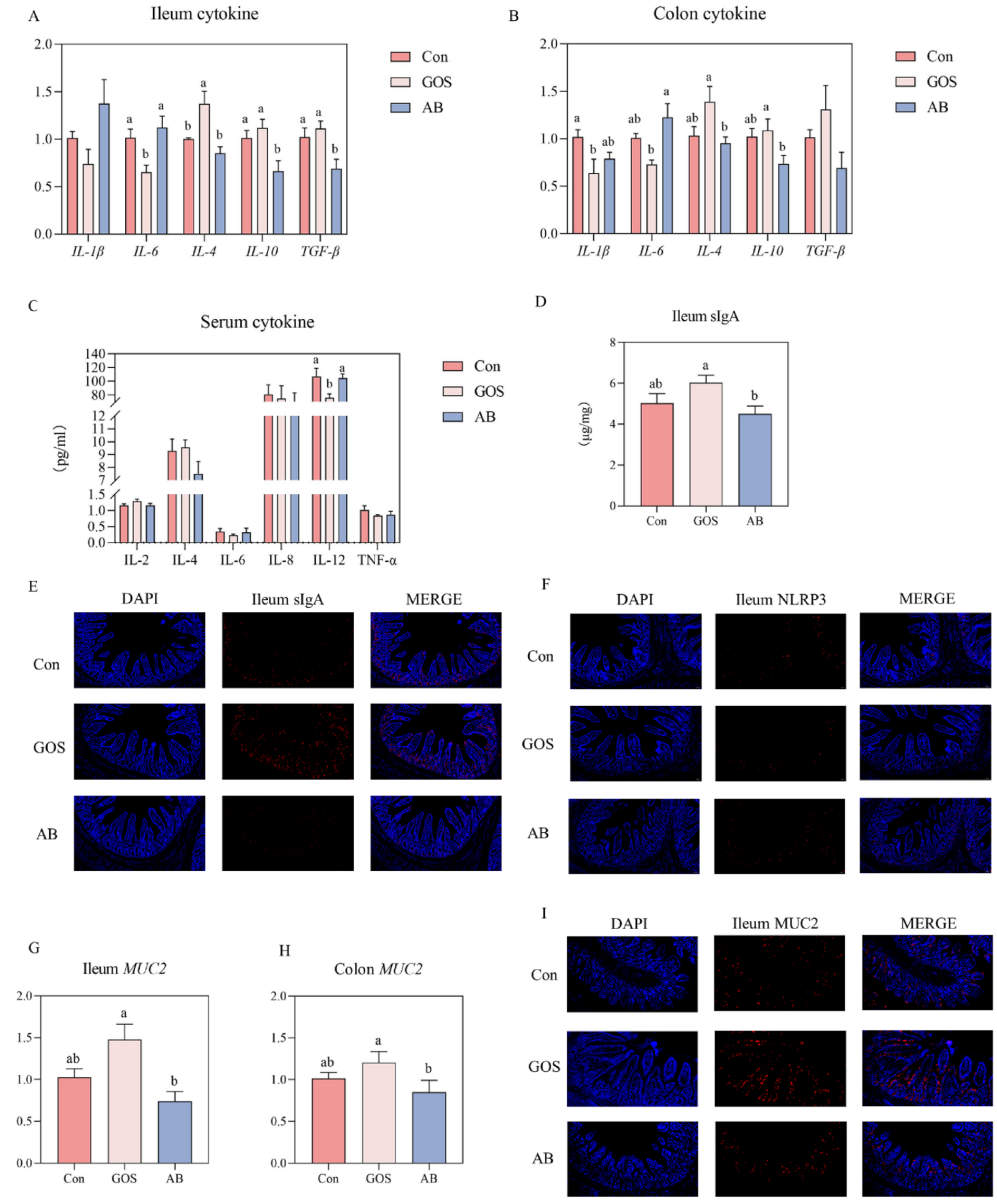
Effect of maternal GOS supplementation on inflammatory profiles, sIgA level and MUC2 expression in piglets. **A** Relative expression level of cytokine mRNA in the ileum mucosa, **B** Relative expression level of cytokine mRNA in the colon mucosa, **C** The content of cytokines in serum. **D** The content of sIgA in the ileum mucosa, **E** Immunofluorescence of sIgA in the ileum, **F** Immunofluorescence of NLRP3 in the ileum **G** Relative expression levels of MUC2 mRNA in the Ileum mucosa, **H** Relative expression level of MUC2 mRNA in the colon mucosa, **I** Immunofluorescence of MUC2 in the ileum (scale bars: 50 μm). Values are presented as means ± SEM, n = 8, One-Way ANOVA, Different lowercase letters indicate significant differences amongst treatment treatments (*P* < 0.05). Con, Basal diet; GOS, Basic diet with 0.5% Galactooligosaccharides (Purity of approximately 70%); AB, Basic diet with 100 ppm chloramphenicol, 20 ppm ciprofloxacin, and 10 ppm vancomycin

The intestinal and serum cytokine levels and sIgA concentration and distribution were showed in Fig. 3A–F. Maternal GOS supplementation significantly decreased the *IL-6* mRNA expressions, while increased the *IL-4* and *IL-10* mRNA expressions in ileum and colon of piglets as compared to those of AB group (*P* < 0.05). The ileal *TGF-β* mRNA expression in AB treatment was lower than that of Con and GOS treatments (*P* < 0.05). The colonic *IL-1β* mRNA expression in GOS treatment was lower than that of Con treatment (*P* < 0.05). As compared to the AB group, GOS treatment increased the sIgA level and fluorescence intensity in ileum of piglets (*P* < 0.05). The difference of NLRP3 fluorescence intensity was not observed in ileum of piglets among three treatments. The mucin layer acts as one of the protective barrier in gut (Fig. 3G–I). The *MUC2* mRNA expressions of ileum and colon in GOS treatment were higher than those of AB treatment (*P* < 0.05). Consistently, the fluorescence intensity of MUC2 in ileum was higher than that of Con and AB treatments (*P* < 0.05).

### Maternal GOS supplementation affected SCFAs levels in intestinal content of piglets

**Fig. 4:**
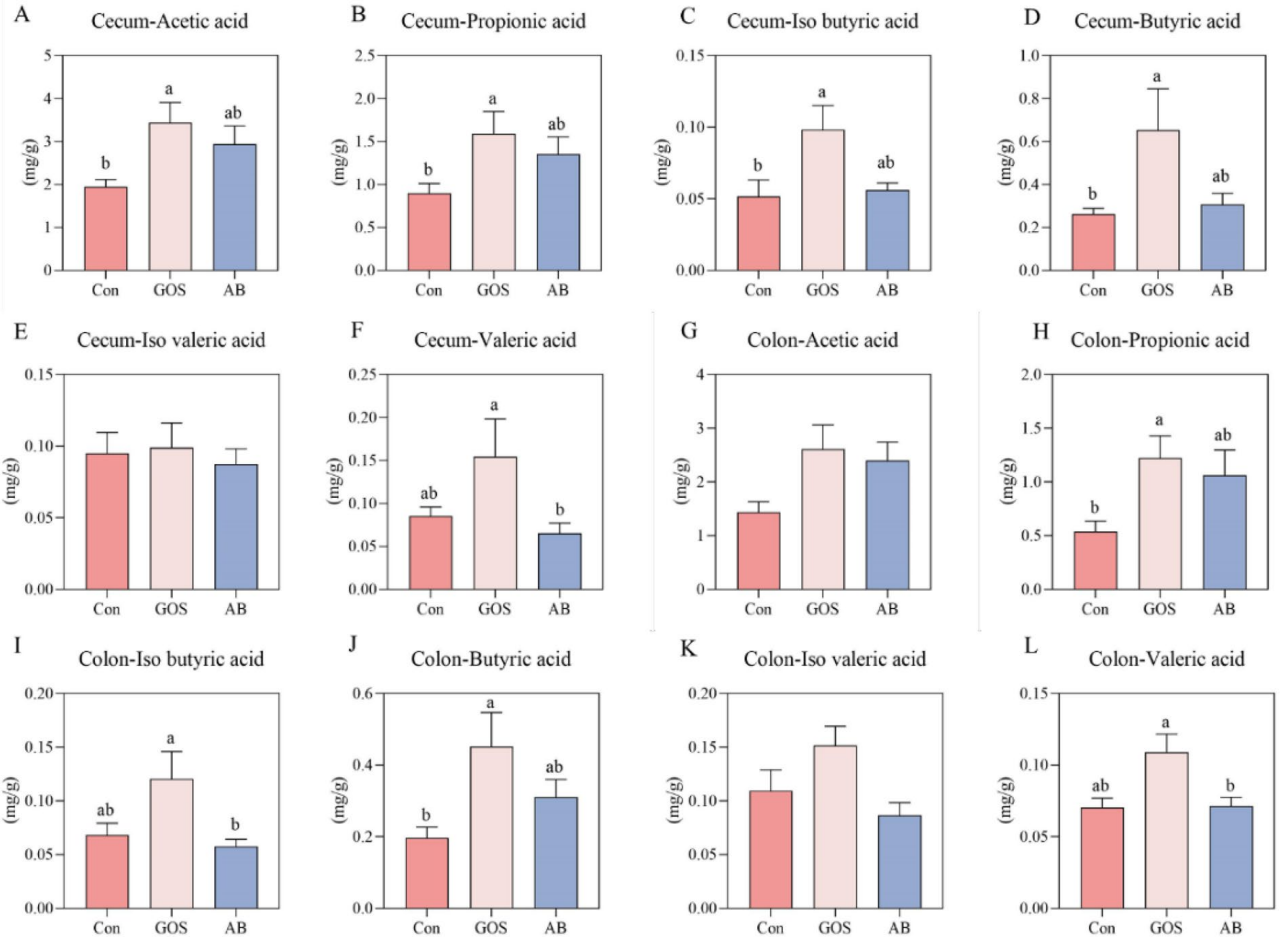
Effects of maternal GOS supplementation on intestinal SCFAs levels of piglets. **A**–**F** Short chain fatty acids in cecal contents (Acetic acid, Propionic acid, Iso butyric acid, Butyric acid, Iso valeric acid, Valeric acid), **G**–**L** Short chain fatty acids in colon contents (Acetic acid, Propionic acid, Iso butyric acid, Butyric acid, Iso valeric acid, Valeric acid). Values are presented as means ± SEM, n = 8, One-Way ANOVA, Different lowercase letters indicate significant differences amongst treatment treatments (*P* < 0.05). Con, Basal diet; GOS, Basic diet with 0.5% Galactooligosaccharides (Purity of approximately 70%); AB, Basic diet with 100 ppm chloramphenicol, 20 ppm ciprofloxacin, and 10 ppm vancomycin

As illustrated in Fig. 4, the concentrations of acetic acid, propionic acid, iso butyric acid, and butyric acid in cecum of piglets from the GOS treatment were significantly higher than those in the Con treatment (*P* < 0.05). The concentration of valeric acid in cecum of piglets in GOS group was higher than that of AB group (*P* < 0.05). The levels of propionic acid and butyric acid in the colon of piglets from the GOS treatment were significantly increased as compared to the Con treatment, and the colonic concentrations of iso butyric acid and valeric acid were notably higher than those in the AB treatment (*P* < 0.05).

### Maternal GOS supplementation altered the gut microbiota composition and structure of piglets

**Fig. 5:**
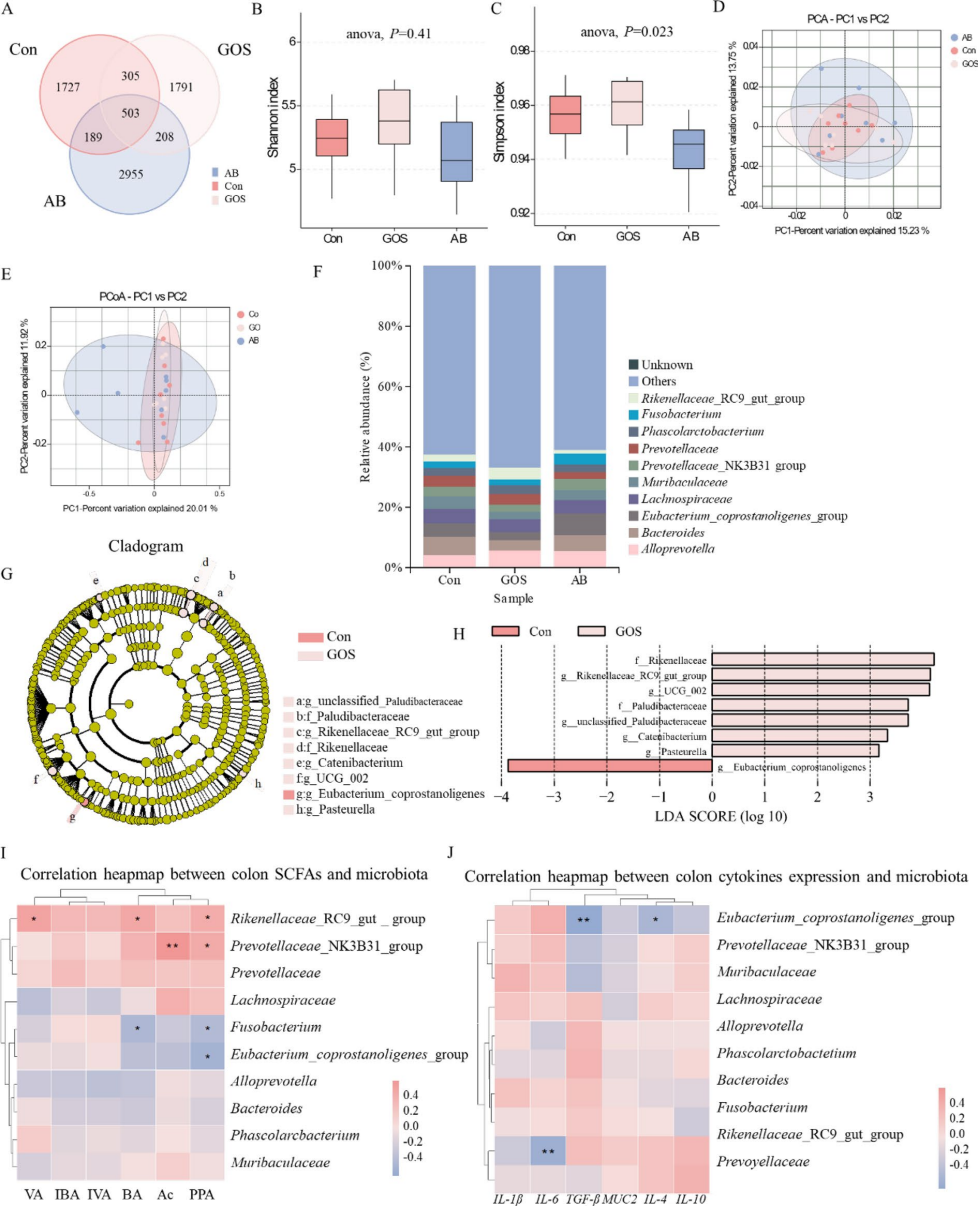
Effect of maternal GOS supplementation on colon microbial composition and structure of piglets. **A** The Venn diagram of the ASVs, **B** Genus level α-Diversity Shannon Index, **C** Genus level α-Diversity Simpson Index, **D** β-Diversity PCA plot, **E** β-Diversity PCoA plot, **F** Genus level species distribution bar chart, **G, H** Genus level LEfSe (LDA score ≥ 3.0) of the microbiota were analyzed, **I** Correlation heatmap between colon SCFAs and microbiota, **J** Correlation heatmap between colon cytokines expression and microbiota. Values are presented as means ± SEM, n = 8, One-Way ANOVA, Different lowercase letters indicate significant differences amongst treatment treatments (*P* < 0.05). Con, Basal diet; GOS, Basic diet with 0.5% Galactooligosaccharides (Purity of approximately 70%); AB, Basic diet with 100 mg/kg chloramphenicol, 20 mg/kg ciprofloxacin, and 10 mg/kg vancomycin

The Venn diagram revealed a total of 7678 OTUs in colon content across the three treatments, with 503 OTUs shared among them. Specifically, the numbers of OTUs in the Con, GOS, and AB treatments are 2724, 2807, and 3855, respectively (Fig. 5A). At the genus level, the Simpson index of colonic microbial α-diversity in the GOS treatment is significantly higher than that in the AB treatment (*P* < 0.05), whereas no significant differences were observed in the Shannon index among three treatments (Fig. 5B, C). PCA or PCoA plots indicated that the community structure of colon microbiota among three treatments were similar (Fig. 5D, E). The species distribution bar chart and LEfSe analysis indicated the variations of colonic microbial composition among the three treatments (Fig. 5F, G). LEfSe analysis (LDA > 3.0) highlighted the enrichment of *Rikenellaceae*_RC9_gut_group in the GOS treatment, while *Fusobacterium* and *Eubacterium*_*coprostanoligenes*_group were enriched in the AB treatment. Additionally, correlation analysis revealed a significant positive correlation between *Rikenellaceae* RC9 and the propionic acid, butyric acid, and valeric acid concentrations in colon, while a negative correlation was observed with colonic IL-6 mRNA expression (*P* < 0.05). Conversely, *Fusobacterium* is significantly negatively correlated with the levels of propionic acid and butyric acid in the colon, and *Eubacterium coprostanoligenes* was negative correlated with colonic propionic acid levels, IL-4 and TGF-β mRNA expression (*P* < 0.05).

## Discussion

Neonatal piglets face distinct health challenges across developmental stages: intrauterine growth restriction due to maternal nutritional status during gestation [46], and impaired gut microbiota colonization coupled with immature immunity during lactation [34]. Maternal dietary fiber supplementation modulates offspring gut microbiota through microbial-host interactions, thereby accelerating intestinal maturation [61]. This study investigated the mechanisms by which maternal GOS supplementation regulates intestinal development and microbiota-host interaction in piglets. Results demonstrated that maternal GOS intervention improved offspring intestinal health by enriching beneficial gut bacteria, enhancing digestive function, strengthening intestinal barrier integrity, and reducing inflammation and diarrhea incidence.

Maternal dietary fiber supplementation can enhance offspring intestinal digestive function, promote metabolism, and improve piglet growth performance, resulting in sustained beneficial effects [14, 25]. Research has demonstrated that the inclusion of functional dietary fibers in gestating sow diets significantly enhances reproductive performance and promotes offspring growth and development. Specifically, supplementation with alfalfa meal, beet pulp, and soybean hulls effectively reduces the incidence of intrauterine growth restriction (IUGR) in piglets [24].And dietary supplementation with inulin- or wheat straw-enriched high-fiber diets significantly enhanced growth velocity in piglets, while concomitantly increasing both weaning weight and litter weight [48, 53]. Tian et al. demonstrated that direct oral administration of GOS during the neonatal period (days 1–7 of lactation) similarly enhanced the average daily gain (ADG) of piglets by the third postnatal week [44]. Consistent with these findings, this study revealed that although the birth weight of piglets in the control group was slightly higher than that of the GOS group, maternal GOS supplementation significantly improved the average daily weight gain and final weight of suckling piglets, greatly enhancing their growth performance. The thymus, as the primary reservoir of T lymphocytes in piglets, regulates innate immune responses and provides protection against pathogen invasion [32]. The liver, as the central organ for metabolism and contains a large number of mitochondria that produce reactive oxygen species, effectively alleviating stress-related issues [59]. Compared to the Con and AB groups, maternal GOS intervention significantly increased the liver and thymus indices of Ningxiang piglets, indicating that the GOS group exhibited stronger immunity, enhanced antioxidant capacity, and greater adaptability to environmental stimuli. Early growth rates in piglets are closely associated with intestinal development and nutritional utilization [52], and diarrhea is more prevalent in suckling piglets with poor intestinal digestive capacity [13]. The ileum, as the longest and thickest segment of the small intestine, possesses a rich capillary network and a well-developed mucosal structure, providing a vast surface area for nutrient absorption and playing a central role in terminal digestion [20, 29]. Digestive enzymes, essential proteins for organismal metabolism, catalyze biochemical reactions for nutrient digestion and metabolism, and their activity serves as a critical indicator of intestinal digestive health [37]. It has been reported that maternal consumption of high-fiber diets during gestation significantly enhances offspring intestinal digestive enzyme activity in swine [22]. In addition, after supplementing newborn piglets with GOS, their intestinal development and digestive function showed phased improvement characteristics: lactase activity significantly increased on the 8th day of lactation, and maltase and sucrase activities also significantly increased at 21 days of age, promoting the efficiency of piglets in decomposing and utilizing carbohydrates [44]. Zhu et al. confirmed that GOS intervention improves the digestive and absorptive functions of piglets through a dual mechanism. On the one hand, it promotes the morphological development of the small intestine, increasing intestinal length to expand the absorptive surface area; on the other hand, it significantly upregulates the expression levels of nutrient transport carriers (such as glucose transporter 2, GLUT2; peptide transporter 1, PEPT1) and digestive enzyme-related genes (aminopeptidase A, APA; aminopeptidase N, APN) in the epithelial cells of the ileum [43].Additionally, given that the acid-binding capacity of the ingested diet exerts a regulatory effect on the gastrointestinal pH, a lower pH level within a certain range can indicate better digestive and absorptive functions, simultaneously providing a favorable environment for the activity of digestive enzymes [17, 38]. Previous study indicates that dietary supplementation with alfalfa and other fermentable fibers significantly enhances gastrointestinal fermentation activity in animals, promotes organic acid production, and reduces digestive tract pH levels. These findings collectively demonstrate the beneficial effects of incorporating fermentable fibers into animal diets [3].The results of the current study found that maternal GOS intervention significantly increased the activities of maltase, α-amylase, and other digestive enzymes in the ileum of piglets, while reducing intestinal pH. These findings confirm that piglets in the GOS group exhibited superior gastrointestinal digestive function, improved nutrient utilization, and a lower incidence of diarrhea, which collectively contribute to their enhanced growth performance.

Optimal villus morphology is also crucial for nutrient intake, with increased villus height and crypt depth favoring nutrient absorption in piglets [28]. Maternal GOS treatment improved the intestinal mucosal morphology and enhanced the tight junction protein ZO-1 and occluding expressions, which might imply superior barrier integrity in piglets. However, serum DAO and D-LA, markers of intestinal barrier integrity [7, 62], were not significant differences among the three maternal interventions. Furthermore, studies suggest that disaccharidase synthesis and secretion reflect intestinal epithelial cells' renewal process [45, 60]. In the present study, the expression of Ki67, a marker of cell proliferation, was higher in GOS treatment. The rapid turnover of intestinal epithelial cells is a crucial condition for maintaining the morphological structure of the gut and also a reliance for the gut to repair damages and maintain effective barrier functions under adverse environments [51]. The secretory mucin MUC2 and immunological components play important roles in the anti-infection of the intestinal barrier. MUC2 produced by goblet cells, can mix with water and electrolytes on the surface of the intestinal mucosa to form a protective barrier [31]. sIgA, produced by B lymphocytes, acts as the primary regulatory factor of the intestinal immune barrier that prevents pathogen colonization and regulates inflammatory responses [2, 55]. Weaning stress elicits disruptions in the glycosylation patterns of MUC and abnormal secretions of immune proteins, leading to impairments in intestinal barrier function and the onset of inflammation [13]. The present study demonstrated that maternal supplementation with GOS effectively elevated the intestinal expression of MUC2 and sIgA of offspring, which might help to enhance the resistance to injury during weaning and alleviate stress symptoms in piglets. Inflammatory cytokines induce and modulate the activation and migration of immune cells, participating in the inflammatory response and serving as sensitive indicators for the occurrence and severity of intestinal inflammation [5]. Pro-inflammatory cytokines such as IL-1β, IL-6, and IFN-γ can disrupt intestinal tight junctions, increase intestinal permeability, and trigger inflammatory reactions [33, 56], while anti-inflammatory cytokines such as IL-10, IL-18, and IL-22 promote intestinal epithelial integrity, control inflammation, and maintain intestinal homeostasis [26, 63]. Maternal GOS treatment enhanced the barrier defense capacity by increasing the intestinal MUC2 and sIgA levels and aiding in alleviating the intestinal inflammation of piglets. On the contrary, the AB treatment group exacerbated intestinal inflammation.

It is reported that maternal GOS supplementation can sustain the stability and superiority of the intestinal microbial structure in piglets [23]. Marzorati M et al. demonstrated that GOS can stimulate the fermentation of *Bifidobacteriaceae* and *Lactobacillaceae*, resulting in the generation of substantial amounts of acetate and propionate, among others [27]. Similarly, in our experiments, the SCFA contents in cecum and colon of piglets from GOS treatment were significantly increased, with particularly notable raises in acetate, propionate and butyrate. SCFAs lead to an acidic gut environment, inhibiting harmful bacteria and opportunistic pathogens [57]. The richness and diversity of intestinal microbiota typically imply a higher-quality microbial structure [6]. In this study, the α-diversity index of colonic microbiota of the GOS treatment piglets was slightly higher, and there were discernible differences in the β-diversity of microbial structures among the treatments. Based on the species distribution bar chart analysis, it was found that *Rikenellaceae* and *Streptococcus* dominated the colons of the GOS treatment piglets. Furthermore, the abundances of beneficial bacteria such as *Lactobacillus*, *Prevotella*, *UCG*-005, and *Muribaculaceae* were significantly higher in the GOS treatment compared to the Con and AB treatments, facilitating better microbial fermentation metabolism and maintenance of intestinal homeostasis [39, 50]. Additionally, although the dominant bacterium *Subdoligranulum* in the Con treatment was positively correlated with High-density lipoprotein cholesterol levels, its function has not yet been elucidated [47]; the predominant bacterium *Eubacterium coprostanoligenes* in the AB treatment could alleviate chemotherapy-induced intestinal mucositis to some extent, but this might be related to the strong inherent resistance of Ningxiang pigs [1]. Analysis of differential bacteria in the colonic microbiota of the three treatments revealed that the predominant bacterium f_*Ruminococcaceae*, which produces short-chain fatty acids (SCFAs), was enriched in the colons of the GOS treatment piglets. In addition, *Prevotella*, *Lactobacillus*, and *Rikenellaceae* enriched in the colons of the GOS treatment piglets were positively correlated with SCFAs. However, AB treatment focused on *Fusobacterium*, which is significantly negatively correlated with SCFAs content. This suggests that maternal GOS supplementation can increase the abundance of beneficial bacteria in the offspring's intestines and support microbial fermentation. Furthermore, the results revealed a strong positive correlation between the expression of MUC2 and the *Rikenellaceae* RC9 gut enriched in the colons of the GOS treatment piglets; it has been previously demonstrated that probiotics can regulate the production of MUC2 and sIgA [9, 42] It was also observed that the *Rikenellaceae* RC9 gut had a strong negative correlation with the expression of the pro-inflammatory cytokine IL-6, but a positive correlation with anti-inflammatory cytokines such as IL-4 and IL-10.

## Conclusions

Maternal GOS supplementation could improve the gut microbiota of piglets, stimulate the development and maturation of the intestinal mucosal barrier, and strengthen the structural and functional integrity of the intestinal mucosal barrier, thereby improving growth performance and reducing the incidence of piglets. This study also presents a substantial data foundation for the ultimate objective of targeting offspring gut health through maternal dietary fiber supplementation during pregnancy and lactation. And underscores the pivotal role of maternal dietary fiber intake in fostering positive gut health outcomes in the subsequent generation.

## Data Availability

No datasets were generated or analysed during the current study.

## References

[1] Bai D, Zhao J, Wang R, Du J, Zhou C, Gu C, Wang Y, Zhang L, Zhao Y, Lu N. Eubacterium coprostanoligenes alleviates chemotherapy-induced intestinal mucositis by enhancing intestinal mucus barrier. Acta Pharm Sin B. 2024;14(4):1677–92. 10.1016/j.apsb.2023.12.015. 38572095 10.1016/j.apsb.2023.12.015 PMC10985029

[2] Bellussi L, Cambi J, Passali D. Functional maturation of nasal mucosa: role of secretory immunoglobulin A (SIgA). Multidiscip Respir Med. 2013;8(1):46. 10.1186/2049-6958-8-46. 23866900 10.1186/2049-6958-8-46 PMC3729437

[3] Brambillasca S, Zunino P, Cajarville C. Addition of inulin, alfalfa and citrus pulp in diets for piglets: influence on nutritional and faecal parameters, intestinal organs, and colonic fermentation and bacterial populations. Livest Sci. 2015;178:243–50.

[4] Castro-Rodríguez DC, Rodríguez-González GL, Menjivar M, Zambrano E. Maternal interventions to prevent adverse fetal programming outcomes due to maternal malnutrition: evidence in animal models. Placenta. 2020;102:49–54. 33218579 10.1016/j.placenta.2020.04.002

[5] de Groot N, Fariñas F, Cabrera-Gómez CG, Pallares FJ, Ramis G. Weaning causes a prolonged but transient change in immune gene expression in the intestine of piglets. J Anim Sci. 2021. 10.1093/jas/skab065. 10.1093/jas/skab065 PMC8051849 33640983

[6] Dou S, Gadonna-Widehem P, Rome V, Hamoudi D, Rhazi L, Lakhal L, Larcher T, Bahi-Jaber N, Pinon-Quintana A, Guyonvarch A, Huërou-Luron IL, Abdennebi-Najar L. Characterisation of early-life fecal microbiota in susceptible and healthy pigs to post-weaning diarrhoea. PLoS ONE. 2017;12(1): e0169851. 10.1371/journal.pone.0169851. 28072880 10.1371/journal.pone.0169851 PMC5225014

[7] Ewaschuk JB, Naylor JM, Zello GA. D-lactate in human and ruminant metabolism. J Nutr. 2005;135(7):1619–25. 10.1093/jn/135.7.1619. 15987839 10.1093/jn/135.7.1619

[8] Ferreira-Maia AP, Matijasevich A, Wang YP. Epidemiology of functional gastrointestinal disorders in infants and toddlers: a systematic review. World J Gastroenterol. 2016;22(28):6547–58. 10.3748/wjg.v22.i28.6547. 27605889 10.3748/wjg.v22.i28.6547 PMC4968134

[9] Fukushima Y, Kawata Y, Hara H, Terada A, Mitsuoka T. Effect of a probiotic formula on intestinal immunoglobulin A production in healthy children. Int J Food Microbiol. 1998;42(1–2):39–44. 10.1016/s0168-1605(98)00056-7. 9706796 10.1016/s0168-1605(98)00056-7

[10] Gao R, Tian S, Wang J, Zhu W. Galacto-oligosaccharides improve barrier function and relieve colonic inflammation via modulating mucosa-associated microbiota composition in lipopolysaccharides-challenged piglets. J Anim Sci Biotechnol. 2021;12:1–16. 34376253 10.1186/s40104-021-00612-z PMC8356462

[11] Gomez-Arango LF, Barrett HL, Wilkinson SA, Callaway LK, McIntyre HD, Morrison M, Dekker Nitert M. Low dietary fiber intake increases Collinsella abundance in the gut microbiota of overweight and obese pregnant women. Gut Microbes. 2018;9(3):189–201. 10.1080/19490976.2017.1406584. 29144833 10.1080/19490976.2017.1406584 PMC6219589

[12] Guan ZW, Yu EZ, Feng Q. Soluble dietary fiber, one of the most important nutrients for the gut microbiota. Molecules. 2021. 10.3390/molecules26226802. 10.3390/molecules26226802 PMC8624670 34833893

[13] Han X, Hu X, Jin W, Liu G. Dietary nutrition, intestinal microbiota dysbiosis and post-weaning diarrhea in piglets. Anim Nutr 2024. 10.1016/j.aninu.2023.12.010 PMC11126776 38800735

[14] He Y, Peng X, Liu Y, Wu Q, Zhou Q, Hu L, Fang Z, Lin Y, Xu S, Feng B, Li J, Zhuo Y, Wu D, Che L. Effects of maternal fiber intake on intestinal morphology, bacterial profile and proteome of newborns using pig as model. Nutrients. 2021;13(1):42. 10.3390/nu13010042 PMC7823571 33375592

[15] He Y, Wang B, Wen L, Wang F, Yu H, Chen D, Su X, Zhang C. Effects of dietary fiber on human health. Food Sci Human Wellness. 2022;11(1):1–10.

[16] Hull HR, Herman A, Gibbs H, Gajewski B, Krase K, Carlson SE, Sullivan DK, Goetz J. The effect of high dietary fiber intake on gestational weight gain, fat accrual, and postpartum weight retention: a randomized clinical trial. BMC Preg Childbirth. 2020;20:1–10. 10.1186/s12884-020-03016-5 PMC7247271 32448177

[17] Huting AM, Middelkoop A, Guan X, Molist F. Using nutritional strategies to shape the gastro-intestinal tracts of suckling and weaned piglets. Animals. 2021;11(2):402. 33562533 10.3390/ani11020402 PMC7914898

[18] Indrio F, Neu J, Pettoello-Mantovani M, Marchese F, Martini S, Salatto A, Aceti A. Development of the gastrointestinal tract in newborns as a challenge for an appropriate nutrition: a narrative review. Nutrients. 2022;14(7):1405. 35406018 10.3390/nu14071405 PMC9002905

[19] Konstantinidis T, Tsigalou C, Karvelas A, Stavropoulou E, Voidarou C, Bezirtzoglou E. Effects of antibiotics upon the gut microbiome: a review of the literature. Biomedicines. 2020. 10.3390/biomedicines8110502. 10.3390/biomedicines8110502 PMC7696078 33207631

[20] Koopmans HS. The role of the ileum in the control of food intake and intestinal adaptation. Can J Physiol Pharmacol. 1990;68(5):650–5. 2340452 10.1139/y90-096

[21] Lei L, Wang Z, Li J, Yang H, Yin Y, Tan B, Chen J. Comparative microbial profiles of colonic digesta between ningxiang pig and large white pig. Animals. 2021. 10.3390/ani11071862. 10.3390/ani11071862 PMC8300102 34201441

[22] Li Y, Zhang L, Liu H, Yang Y, He J, Cao M, Yang M, Zhong W, Lin Y, Zhuo Y, Fang Z, Che L, Feng B, Xu S, Li J, Zhao X, Jiang X, Wu D. Effects of the ratio of insoluble fiber to soluble fiber in gestation diets on sow performance and offspring intestinal development. Animals. 2019. 10.3390/ani9070422. 10.3390/ani9070422 PMC6680925 31284518

[23] Lim JA, Cha J, Choi S, Kim JH, Kim D. Early colonization of the intestinal microbiome of neonatal piglets is influenced by the maternal microbiome. Animals. 2023. 10.3390/ani13213378. 10.3390/ani13213378 PMC10650534 37958132

[24] Liu B, Zhu X, Cui Y, Wang W, Liu H, Li Z, Guo Z, Ma S, Li D, Wang C, Shi Y. Consumption of dietary fiber from different sources during pregnancy alters sow gut microbiota and improves performance and reduces inflammation in sows and piglets. mSystems. 2021. 10.1128/mSystems.00591-20. 10.1128/mSystems.00591-20 PMC7842364 33500330

[25] Liu X, Wei X, Feng Y, Liu H, Tang J, Gao F, Shi B. Supplementation with complex dietary fiber during late pregnancy and lactation can improve progeny growth performance by regulating maternal antioxidant status and milk quality. Antioxidants. 2023. 10.3390/antiox13010022. 10.3390/antiox13010022 PMC10812556 38275642

[26] Macia L, Tan J, Vieira AT, Leach K, Stanley D, Luong S, Maruya M, Ian McKenzie C, Hijikata A, Wong C, Binge L, Thorburn AN, Chevalier N, Ang C, Marino E, Robert R, Offermanns S, Teixeira MM, Moore RJ, Flavell RA, Fagarasan S, Mackay CR. Metabolite-sensing receptors GPR43 and GPR109A facilitate dietary fibre-induced gut homeostasis through regulation of the inflammasome. Nat Commun. 2015;6:6734. 10.1038/ncomms7734. 25828455 10.1038/ncomms7734

[27] Marzorati M, Ghyselinck J, Van den Abbeele P, Maruszak A, Harthoorn L. Galactooligosaccharide (GOS) reduces branched short-chain fatty acids, ammonium, and pH in a short-term colonic fermentation model. Appl Microbiol. 2023;3(1):90–103.

[28] Montagne L, Pluske J, Hampson D. A review of interactions between dietary fibre and the intestinal mucosa, and their consequences on digestive health in young non-ruminant animals. Anim Feed Sci Technol. 2003;108(1–4):95–117.

[29] Nigam Y, Knight J, Williams N. Gastrointestinal tract 4: anatomy and role of the jejunum and ileum. Nurs times. 2019;115(9):43–6.

[30] Omoto T, Kyozuka H, Murata T, Fukuda T, Isogami H, Okoshi C, Yasuda S, Yamaguchi A, Sato A, Ogata Y, Shinoki K, Hosoya M, Yasumura S, Hashimoto K, Nishigori H, Fujimori K. Association between preconception dietary fiber intake and hypertensive disorders of pregnancy: the Japan Environment and Children’s Study. Preg Hypertension. 2024;37: 101139. 10.1016/j.preghy.2024.101139. 10.1016/j.preghy.2024.101139 38878601

[31] Paone P, Cani PD. Mucus barrier, mucins and gut microbiota: The expected slimy partners? Gut. 2020;69(12):2232–43. 10.1136/gutjnl-2020-322260. 32917747 10.1136/gutjnl-2020-322260 PMC7677487

[32] Pearse G. Normal structure, function and histology of the thymus. Toxicol Pathol. 2006;34(5):504–14. 10.1080/01926230600865549. 17067941 10.1080/01926230600865549

[33] Pié S, Lallès JP, Blazy F, Laffitte J, Sève B, Oswald IP. Weaning is associated with an upregulation of expression of inflammatory cytokines in the intestine of piglets. J Nutr. 2004;134(3):641–7. 10.1093/jn/134.3.641. 14988461 10.1093/jn/134.3.641

[34] Rahman MT, Brown-Brandl TM, Rohrer GA, Sharma SR, Manthena V, Shi Y. Statistical and machine learning approaches to describe factors affecting preweaning mortality of piglets. Transl Anim Sci. 2023;7(1):txad117. 10.1093/tas/txad117. 38034395 10.1093/tas/txad117 PMC10684129

[35] Ramirez J, Guarner F, Bustos Fernandez L, Maruy A, Sdepanian VL, Cohen H. Antibiotics as major disruptors of gut microbiota. Front Cell Infect Microbiol. 2020;10: 572912. 10.3389/fcimb.2020.572912. 33330122 10.3389/fcimb.2020.572912 PMC7732679

[36] Shang Q, Liu S, Liu H, Mahfuz S, Piao X. Maternal supplementation with a combination of wheat bran and sugar beet pulp during late gestation and lactation improves growth and intestinal functions in piglets. Food Funct. 2021;12(16):7329–42. 10.1039/d1fo00014d. 34179915 10.1039/d1fo00014d

[37] Shang Q, Ma X, Liu H, Liu S, Piao X. Effect of fibre sources on performance, serum parameters, intestinal morphology, digestive enzyme activities and microbiota in weaned pigs. Arch Anim Nutr. 2020;74(2):121–37. 10.1080/1745039x.2019.1684148. 31821028 10.1080/1745039X.2019.1684148

[38] Shi Z, Wang T, Kang J, Li Y, Li Y, Xi L. Effects of weaning modes on the intestinal ph, activity of digestive enzymes, and intestinal morphology of piglets. Animals. 2022;12(17):2200. 36077920 10.3390/ani12172200 PMC9455048

[39] Smith BJ, Miller RA, Schmidt TM. Muribaculaceae genomes assembled from metagenomes suggest genetic drivers of differential response to acarbose treatment in mice. mSphere. 2021;6(6):e0085121. 10.1128/msphere.00851-21. 34851167 10.1128/msphere.00851-21 PMC8636109

[40] Sun C, Song R, Zhou J, Jia Y, Lu J. Fermented bamboo fiber improves productive performance by regulating gut microbiota and inhibiting chronic inflammation of sows and piglets during late gestation and lactation. Microbiol Spectr. 2023;11(3): e0408422. 10.1128/spectrum.04084-22. 37042787 10.1128/spectrum.04084-22 PMC10269633

[41] Sun J, Wang J, Ma W, Miao M, Sun G. Effects of additional dietary fiber supplements on pregnant women with gestational diabetes: a systematic review and meta-analysis of randomized controlled studies. Nutrients. 2022. 10.3390/nu14214626. 10.3390/nu14214626 PMC9658588 36364883

[42] Teng T, Gao F, He W, Fu H, Guo J, Bai G, Shi B. An early fecal microbiota transfer improves the intestinal conditions on microflora and immunoglobulin and antimicrobial peptides in piglets. J Agric Food Chem. 2020;68(17):4830–43. 10.1021/acs.jafc.0c00545. 32252520 10.1021/acs.jafc.0c00545

[43] Tian S, Wang J, Gao R, Wang J, Zhu W. Early-life galacto-oligosaccharides supplementation alleviates the small intestinal oxidative stress and dysfunction of lipopolysaccharide-challenged suckling piglets. J Anim Sci Biotechnol. 2022;13(1):70. 10.1186/s40104-022-00711-5. 35655292 10.1186/s40104-022-00711-5 PMC9164537

[44] Tian S, Wang J, Yu H, Wang J, Zhu W. Effects of galacto-oligosaccharides on growth and gut function of newborn suckling piglets. J Anim Sci Biotechnol. 2018;9:75. 10.1186/s40104-018-0290-9. 30349690 10.1186/s40104-018-0290-9 PMC6193306

[45] Tsukahara T, Inoue R, Nakatani M, Fukuta K, Kishino E, Ito T, Ushida K. Influence of weaning age on the villous height and disaccharidase activities in the porcine small intestine. Anim Sci J. 2016;87(1):67–75. 10.1111/asj.12399. 26153481 10.1111/asj.12399

[46] Van Ginneken C, Ayuso M, Van Bockstal L, Van Cruchten S. Preweaning performance in intrauterine growth-restricted piglets: characteristics and interventions. Mol Reprod Dev. 2023;90(7):697–707. 10.1002/mrd.23614. 35652465 10.1002/mrd.23614

[47] Van Hul M, Le Roy T, Prifti E, Dao MC, Paquot A, Zucker JD, Delzenne NM, Muccioli G, Clément K, Cani PD. From correlation to causality: the case of Subdoligranulum. Gut Microbes. 2020;12(1):1–13. 10.1080/19490976.2020.1849998. 10.1080/19490976.2020.1849998 PMC7744154 33323004

[48] Veum TL, Crenshaw JD, Crenshaw TD, Cromwell GL, Easter RA, Ewan RC, Nelssen JL, Miller ER, Pettigrew JE, Ellersieck MR. The addition of ground wheat straw as a fiber source in the gestation diet of sows and the effect on sow and litter performance for three successive parities. J Anim Sci. 2009;87(3):1003–12. 10.2527/jas.2008-1119. 18952734 10.2527/jas.2008-1119

[49] Wang J, Tian S, Yu H, Wang J, Zhu W. Response of colonic mucosa-associated microbiota composition, mucosal immune homeostasis, and barrier function to early life galactooligosaccharides intervention in suckling piglets. J Agric Food Chem. 2019;67(2):578–88. 10.1021/acs.jafc.8b05679. 30562014 10.1021/acs.jafc.8b05679

[50] Wang L, Liu Q, Chen Y, Zheng X, Wang C, Qi Y, Dong Y, Xiao Y, Chen C, Chen T, Huang Q, Zhai Z, Long C, Yang H, Li J, Wang L, Zhang G, Liao P, Liu YX, Huang P, Huang J, Wang Q, Chu H, Yin J, Yin Y. Antioxidant potential of Pediococcus pentosaceus strains from the sow milk bacterial collection in weaned piglets. Microbiome. 2022;10(1):83. 10.1186/s40168-022-01278-z. 35650642 10.1186/s40168-022-01278-z PMC9158380

[51] Wang M, Wang L, Tan X, Wang L, Xiong X, Wang Y, Wang Q, Yang H, Yin Y. The developmental changes in intestinal epithelial cell proliferation, differentiation, and shedding in weaning piglets. Anim Nutr. 2022;9:214–22. 35600553 10.1016/j.aninu.2021.11.006 PMC9092860

[52] Wang M, Yang C, Wang Q, Li J, Li Y, Ding X, Yin J, Yang H, Yin Y. The growth performance, intestinal digestive and absorptive capabilities in piglets with different lengths of small intestines. Animal. 2020;14(6):1196–203. 31829913 10.1017/S175173111900288X

[53] Wang YS, Zhou P, Liu H, Li S, Zhao Y, Deng K, Cao DD, Che LQ, Fang ZF, Xu SY, Lin Y, Feng B, Li J, Wu D. Effects of inulin supplementation in low- or high-fat diets on reproductive performance of sows and antioxidant defence capacity in sows and offspring. Reprod Domest Anim. 2016;51(4):492–500. 10.1111/rda.12707. 27174736 10.1111/rda.12707

[54] Wells JM, Gao Y, de Groot N, Vonk MM, Ulfman L, van Neerven RJJ. Babies, bugs, and barriers: dietary modulation of intestinal barrier function in early life. Annu Rev Nutr. 2022;42:165–200. 10.1146/annurev-nutr-122221-103916. 35697048 10.1146/annurev-nutr-122221-103916

[55] Willing BP, Russell SL, Finlay BB. Shifting the balance: antibiotic effects on host-microbiota mutualism. Nat Rev Microbiol. 2011;9(4):233–43. 10.1038/nrmicro2536. 21358670 10.1038/nrmicro2536

[56] Xia B, Wu W, Zhang L, Wen X, Xie J, Zhang H. Gut microbiota mediates the effects of inulin on enhancing sulfomucin production and mucosal barrier function in a pig model. Food Funct. 2021;12(21):10967–82. 10.1039/d1fo02582a. 34651635 10.1039/d1fo02582a

[57] Yamamura R, Inoue KY, Nishino K, Yamasaki S. Intestinal and fecal pH in human health. Front Microbiomes. 2023;2:1192316. 10.3389/frmbi.2023.1192316 PMC12993532 41853365

[58] Yin L, Yang H, Li J, Li Y, Ding X, Wu G, Yin Y. Pig models on intestinal development and therapeutics. Amino Acids. 2017;49(12):2099–106. 10.1007/s00726-017-2497-z. 28986749 10.1007/s00726-017-2497-z

[59] Yu L, Li H, Peng Z, Ge Y, Liu J, Wang T, Wang H, Dong L. Early weaning affects liver antioxidant function in piglets. Animals. 2021;11(9):2679. 34573645 10.3390/ani11092679 PMC8469846

[60] YunXian L, FeiYun Y, Chao W, JinXiu H, ZuoHua L. Research on the development and influence of disaccharidase activity in the smallintestinal mucosa in Rongchang piglets. Heilongjiang Anim Sci Vet Med. 2011. 10.13881/j.cnki.hljxmsy.2011.17.029.

[61] Zaidi AZ, Moore SE, Okala SG. Impact of maternal nutritional supplementation during pregnancy and lactation on the infant gut or breastmilk microbiota: a systematic review. Nutrients. 2021;13(4):1137. 33808265 10.3390/nu13041137 PMC8067242

[62] Zhang L, Fan X, Zhong Z, Xu G, Shen J. Association of plasma diamine oxidase and intestinal fatty acid-binding protein with severity of disease in patient with heat stroke. Am J Emerg Med. 2015;33(7):867–71. 10.1016/j.ajem.2015.01.047. 25913083 10.1016/j.ajem.2015.01.047

[63] Zhang Y, Dong A, Xie K, Yu Y. Dietary supplementation with high fiber alleviates oxidative stress and inflammatory responses caused by severe sepsis in mice without altering microbiome diversity. Front Physiol. 2018;9:1929. 10.3389/fphys.2018.01929. 30713502 10.3389/fphys.2018.01929 PMC6345681

